# n–n ZnO–Ag_2_CrO_4_ heterojunction photoelectrodes with enhanced visible-light photoelectrochemical properties

**DOI:** 10.1039/c9ra00639g

**Published:** 2019-03-11

**Authors:** Mahsa Pirhashemi, Sami Elhag, Rania E. Adam, Aziz Habibi-Yangjeh, Xianjie Liu, Magnus Willander, Omer Nur

**Affiliations:** Department of Science and Technology (ITN), Linköping University Campus Norrköping 60174 Norrköping Sweden sami.elhag@liu.se; University of Mohaghegh Ardabili Iran; Department of Physics, Chemistry, and Biology (IFM), Linköping University 58183 Linköping Sweden

## Abstract

In this study, ZnO nanorods (NRs) were hydrothermally grown on an Au-coated glass substrate at a relatively low temperature (90 °C), followed by the deposition of Ag_2_CrO_4_ particles *via* a successive ionic layer adsorption and reaction (SILAR) route. The content of the Ag_2_CrO_4_ particles on ZnO NRs was controlled by changing the number of SILAR cycles. The fabricated ZnO–Ag_2_CrO_4_ heterojunction photoelectrodes were subjected to morphological, structural, compositional, and optical property analyses; their photoelectrochemical (PEC) properties were investigated under simulated solar light illumination. The photocurrent responses confirmed that the ability of the ZnO–Ag_2_CrO_4_ heterojunction photoelectrodes to separate the photo-generated electron–hole pairs is stronger than that of bare ZnO NRs. Impressively, the maximum photocurrent density of about 2.51 mA cm^−2^ at 1.23 V (*vs.* Ag/AgCl) was measured for the prepared ZnO–Ag_2_CrO_4_ photoelectrode with 8 SILAR cycles (denoted as ZnO–Ag_2_CrO_4_-8), which exhibited about 3-fold photo-enhancement in the current density as compared to bare ZnO NRs (0.87 mA cm^−2^) under similar conditions. The improvement in photoactivity was attributed to the ideal band gap and high absorption coefficient of the Ag_2_CrO_4_ particles, which resulted in improved solar light absorption properties. Furthermore, an appropriate annealing treatment was proven to be an efficient process to increase the crystallinity of Ag_2_CrO_4_ particles deposited on ZnO NRs, which improved the charge transport characteristics of the ZnO–Ag_2_CrO_4_-8 photoelectrode annealed at 200 °C and increased the performance of the photoelectrode. The results achieved in the present work present new insights for designing n–n heterojunction photoelectrodes for efficient and cost-effective PEC applications and solar-to-fuel energy conversions.

## Abstract 2

ZnO NRs hydrothermally grown on Au coated glass substrate, followed by deposition of Ag_2_CrO_4_ particles *via* SILAR route. The content of the Ag_2_CrO_4_ particles on the ZnO NRs were controlled by changing the number of SILAR cycles.

## Graphical abstract

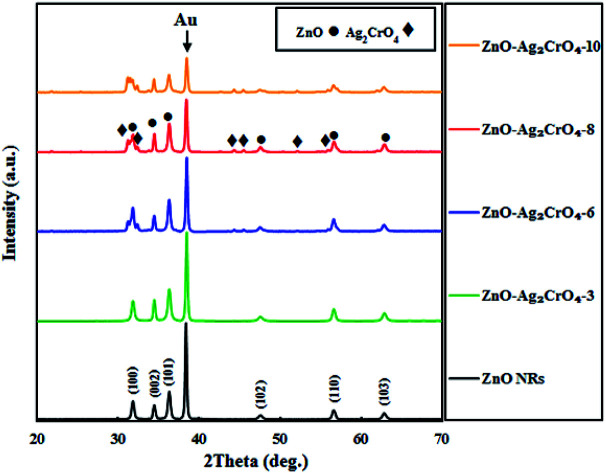

## Introduction

The greatest challenge in the current society is the reduction in the amount of environmental pollution and the dependence on fossil fuels. In particular, fossil fuels are still the main energy resources for humanity. In addition to the fact that fossil fuels will be depleted in the future, the consumption of fossil fuels boosts CO_2_ emissions, which exacerbates global warming. Hence, there is an urgent need to find a viable alternative for fossil fuels and improve the harvesting of green and clean energy.^1–3^

A photoelectrochemical (PEC) process based on semiconductor materials for fuel generation through water splitting offers a versatile strategy to develop an energy conversion device by utilizing the solar energy to carry out the required electrochemical reactions to produce clean energy.^4,5^ In fact, the PEC cells based on metal oxide semiconductors such as TiO_2_ and ZnO have attracted considerable attention as effective photoelectrodes due to their good chemical stability, excellent electron mobility, environment-friendly features, and low price for photochemical water splitting.^6–8^ Nevertheless, the large band gap of ZnO limits its visible-light response. In addition, the rapid recombination of photoinduced e^−^/h^+^ pairs strongly lowers the photoconversion efficiency.^9,10^ To overcome these difficulties, the constructed heterojunction between ZnO and narrow-band-gap semiconductors with appropriate energy levels can not only broaden the light absorption region but also facilitate the separation and transfer of photocarriers.^11–15^ In particular, nanostructure composites containing n–n heterojunctions with direct contact between two n-type semiconductors have widespread potential applications because of the formed electric field produced at the junction, resulting in efficient charge separation, as demonstrated in InN/ZnO,^16^ Fe_2_O_3_/ZnO,^17^ BiVO_4_/P25,^18^ CdWO_4_/Bi_2_O_2_CO_3_ (ref. 19) and ZnO/Ag_3_VO_4_.^20^

Silver-based semiconductors have currently attracted extensive research attention due to their electronic and crystalline structures and suitable band gaps.^21^ Most recently, special attention has been paid to silver chromate (Ag_2_CrO_4_) as an important candidate for combination with ZnO since it offers an appropriate band gap (*e.g.*, ∼1.8 eV), which is favorable for the utilization of a significant portion of the solar energy.^22,23^ However, the photostability of pure Ag_2_CrO_4_ is relatively low. Hence, many Ag_2_CrO_4_-based nanocomposites such as WO_3_/Ag_2_CrO_4_,^24^ g-C_3_N_4_/Ag_2_CrO_4_,^25^ TiO_2_/Ag_2_CrO_4_,^26^ and In_2_S_3_/Ag_2_CrO_4_ (ref. 27) have been prepared and have demonstrated improved stability and photocatalytic performance when compared with pure Ag_2_CrO_4_. As proven in our previous work, ZnO–Ag_2_CrO_4_ nanocomposites exhibit excellent performance in dye photodegradation under visible-light illumination.^22^ It is notable that most of the above-mentioned studies have focused on the photocatalytic applications. Up to now, there have been no reports on the Ag_2_CrO_4_ sensitization of ZnO nanorods (NRs) as the PEC photoelectrode in the water splitting system. In this direction, the incorporation of n-type Ag_2_CrO_4_ with n-type ZnO to form an n–n heterojunction is expected to have enhanced PEC efficiency for water splitting applications due to higher absorption of solar energy and considerable retardation of e^−^/h^+^ pairs from undesirable recombination.^28,29^

In this work, we demonstrated a facile growth process to obtain ZnO NRs on an Au-coated glass substrate with a high yield at a low temperature. Subsequently, Ag_2_CrO_4_ nanoparticles were integrated on the surface of ZnO NRs by successive ionic layer adsorption and reaction (SILAR) to form ZnO–Ag_2_CrO_4_ heterojunction photoelectrodes, followed by annealing. Compared with the pristine ZnO NR photoelectrode, the ZnO–Ag_2_CrO_4_ heterojunction exhibited significant optoelectronic properties including high photocurrent/responsivity and a short response time. In addition, the ZnO–Ag_2_CrO_4_ heterojunction photoelectrodes with different SILAR cycles of Ag_2_CrO_4_ annealed at various temperatures were studied. Our PEC performances were comparable to those of many metal oxide-based photoanodes in recent reports.^30–32^ In addition, the mechanism for the enhanced PEC performance on the ZnO–Ag_2_CrO_4_ heterojunction photoelectrodes was discussed in detail.

## Experimental

### Materials

All chemicals used in this study were of analytical grade (Sigma-Aldrich), and they were used without any further purification. Distilled water was used throughout this experiment.

### Synthetic procedures

#### Growth of ZnO NRs on Au-coated glass

Following a previously reported method,^33^ ZnO NRs were grown on an Au-coated glass by a seed-assisted hydrothermal method. For this purpose, the ZnO seed layer was deposited on the cleaned Au-coated glass with the aid of a spin coating technique at 500 and 3000 rpm for 5 and 20 s, respectively; this process was repeated 3 times. The ZnO seed precursor was prepared by adding 0.03 M potassium hydroxide solution in methanol dropwise into a 0.01 M zinc acetate dehydrate solution in methanol under magnetic stirring at 60 °C for 2 h. Afterwards, the seeded substrates were fixed upside down in a Teflon sample holder and dipped horizontally into a mixed aqueous solution containing 0.05 M zinc nitrate hexahydrate (Zn(NO_3_)_2_·6H_2_O) and 0.05 M hexamethylenetetramine; then, it was heated for 5 h at 90 °C in an oven. Finally, the samples were washed with distilled water and dried under nitrogen gas.

#### Fabrication of the ZnO–Ag_2_CrO_4_ photoelectrode

**Scheme 1:**
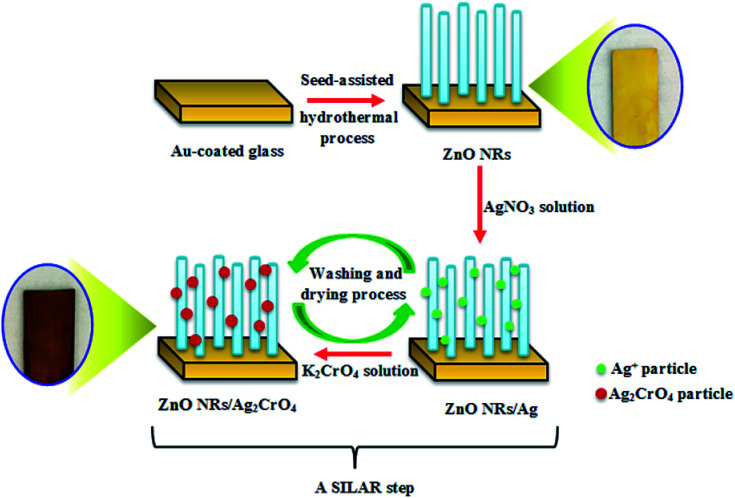
Schematic illustration of the preparation procedure for the ZnO–Ag_2_CrO_4_ photoelectrodes.

In order to deposit Ag_2_CrO_4_ nanoparticles over the ZnO NR photoelectrode, a SILAR-assisted annealing process was implemented. In this method, ZnO NRs were immersed in two different solutions sequentially; first, the sample was immersed in silver nitrate with 0.05 M in methanol/water (3 : 1/v : v) for 2 min. Afterwards, the excess reagent was removed by washing with methanol and dried in an N_2_ stream. Second, the sample was immersed in sodium chromate (Na_2_CrO_4_) with 0.05 M in methanol/water (3 : 1/v : v) for 2 min. Subsequently, the sample was washed and dried again. These sequential immersion steps proceeding at room temperature were termed as one SILAR cycle. This procedure was repeated for 3, 6, 8, and 10 cycles, producing dark purple samples (Scheme 1). Finally, the resulting samples were additionally dried for 1 h at 60 °C. The as-prepared photoelectrodes were denoted as ZnO–Ag_2_CrO_4_-*n*, where *n* represents the number of SILAR cycles.

### Characterization methods

A field emission scanning electron microscope (FESEM, Quanta 200 FEG) was used to observe the morphology of the samples. The crystallinity of the prepared samples was acquired using an X-ray diffractometer (Shimadzu Lab-X XRD-6000) with Cu Kα. X-ray photoelectron spectroscopy (XPS, PHI 5600 mode) was carried out for the surface chemistry analysis. The binding energies were corrected by using the contaminant carbon (C 1s) with binding energy of 284.6 eV. Light absorption properties were measured using a UV-Vis DRS (JASCO, UV-550) spectrophotometer.

### PEC measurements

The PEC properties and Mott–Schottky plots of the fabricated photoelectrodes were studied with a three-electrode configuration using a potentiostat (SP-200, Bio-Logic, Claix, France). The as-fabricated photoelectrodes were applied as working electrodes, whereas an Ag/AgCl/KCl (3 M) electrode and a Pt sheet were used as the reference and counter electrodes, respectively. The PEC measurements were obtained under simulated solar light illumination (AM 1.5G, LCS-100, Newport, model 94011A), while the photoelectrode with 1 cm × 1 cm area was submerged in 0.1 M aqueous Na_2_SO_4_ solution as the electrolyte.

## Result and discussion

**Fig. 1:**
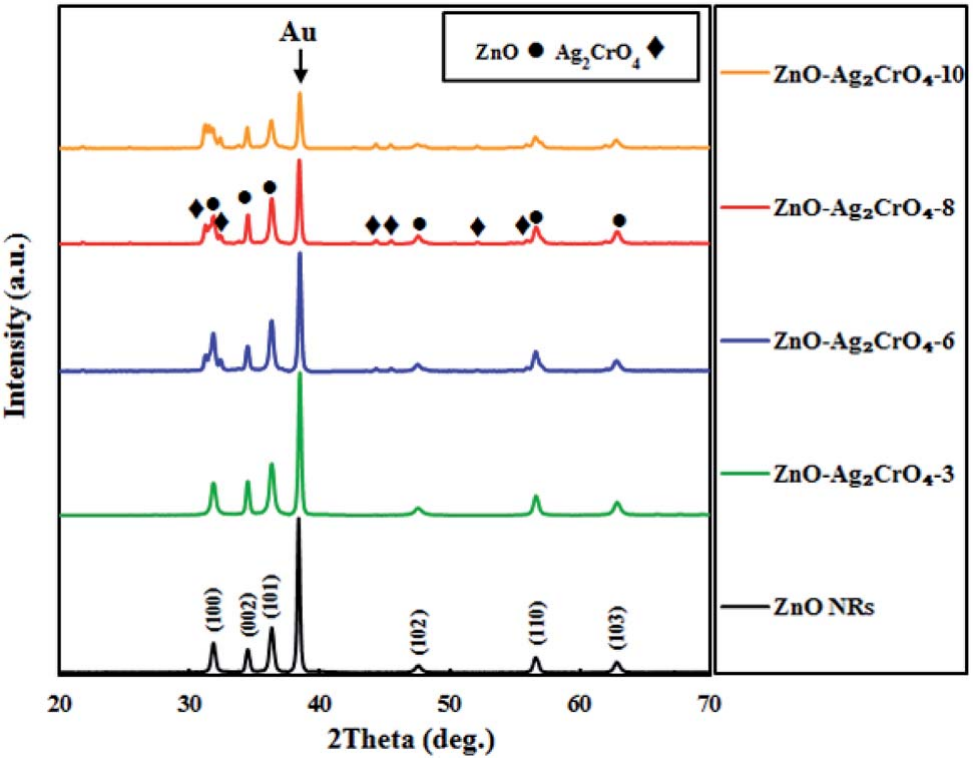
XRD patterns of ZnO NRs and ZnO–Ag_2_CrO_4_ photoelectrodes with different numbers of SILAR cycles.

The phase structures of the ZnO NR and ZnO–Ag_2_CrO_4_ heterostructures with different SILAR cycles were explored by XRD patterns, as displayed in Fig. 1. The peak located at 2*θ* = 38.22° for all samples is assigned to the Au-coated glass substrate.^34^ It is evident that the XRD pattern of ZnO NRs coincides well with that of the hexagonal wurtzite structure (JCPDS no. 36-1451).^35^ The XRD patterns of the heterojunctions revealed the presence of wurtzite ZnO NRs along with monoclinic Ag_2_CrO_4_ (JCPDS no. 26-0952),^22^ further indicating that the Ag_2_CrO_4_ nanoparticles were successfully deposited on the surface of ZnO NRs. Moreover, no peaks for undesirable materials were observed, which demonstrated the high purity of the samples.

**Fig. 2:**
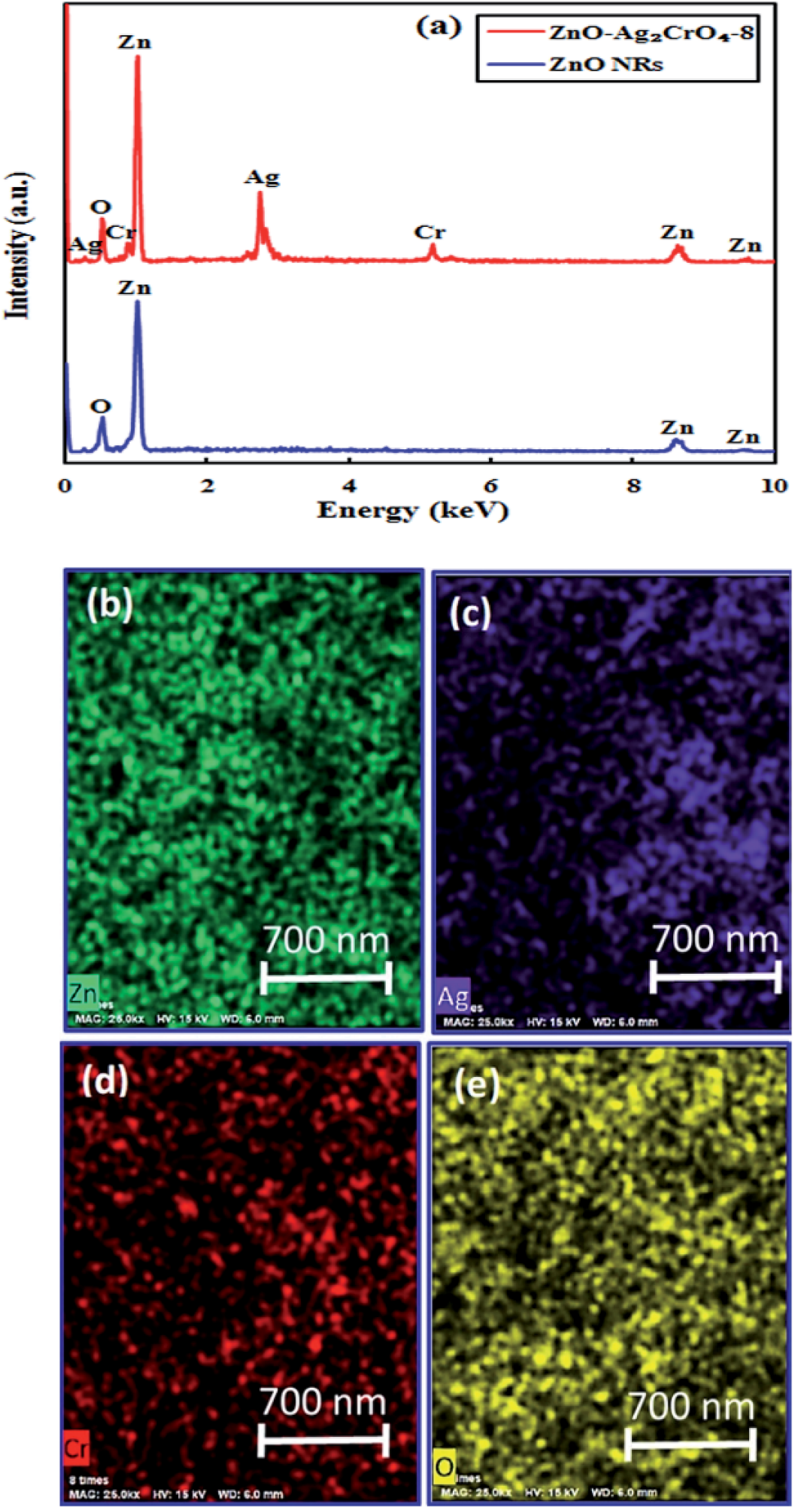
(a) EDX spectra for ZnO NRs and ZnO–Ag_2_CrO_4_-8 samples. (b–e) EDX mapping of the ZnO–Ag_2_CrO_4_-8 photoelectrode.

In order to obtain the elemental information, EDX spectroscopy was utilized for ZnO NRs and ZnO–Ag_2_CrO_4_-8 heterostructures. As revealed in Fig. 2(a), Zn and O peaks result from ZnO NRs. It is clearly illustrated that Zn, Ag, Cr, and O elements distinctly co-exist in the prepared ZnO–Ag_2_CrO_4_-8 photoelectrode. In addition, it can be clearly revealed from Fig. 2(b–e) that all the elements are distributed homogeneously in the ZnO–Ag_2_CrO_4_-8 heterostructures, confirming that Ag_2_CrO_4_ not only successfully combined with ZnO NRs, but was also well dispersed on ZnO NRs.

**Fig. 3:**
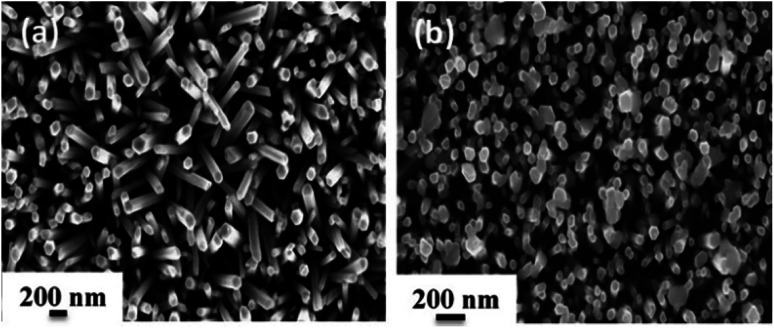
The FESEM images of (a) ZnO NRs and (b) ZnO–Ag_2_CrO_4_-8 photoelectrodes.

In order to explore the morphology and structure of the prepared samples, FE-SEM analysis was carried out. As illustrated in Fig. 3, ZnO NRs with a typical hexagonal structure have a relatively uniform diameter of about 100 nm, and they grow vertically on the Au-coated glass substrate with an average height of 1.3–1.8 μm. When the SILAR process was applied to prepare Ag_2_CrO_4_ structures, sphere-like Ag_2_CrO_4_ nanoparticles with diameters less than 200 nm were dispersed on the surface of ZnO NRs (Fig. 3(b)). These spherical particles are not only visible on the top but also between ZnO NRs.

**Fig. 4:**
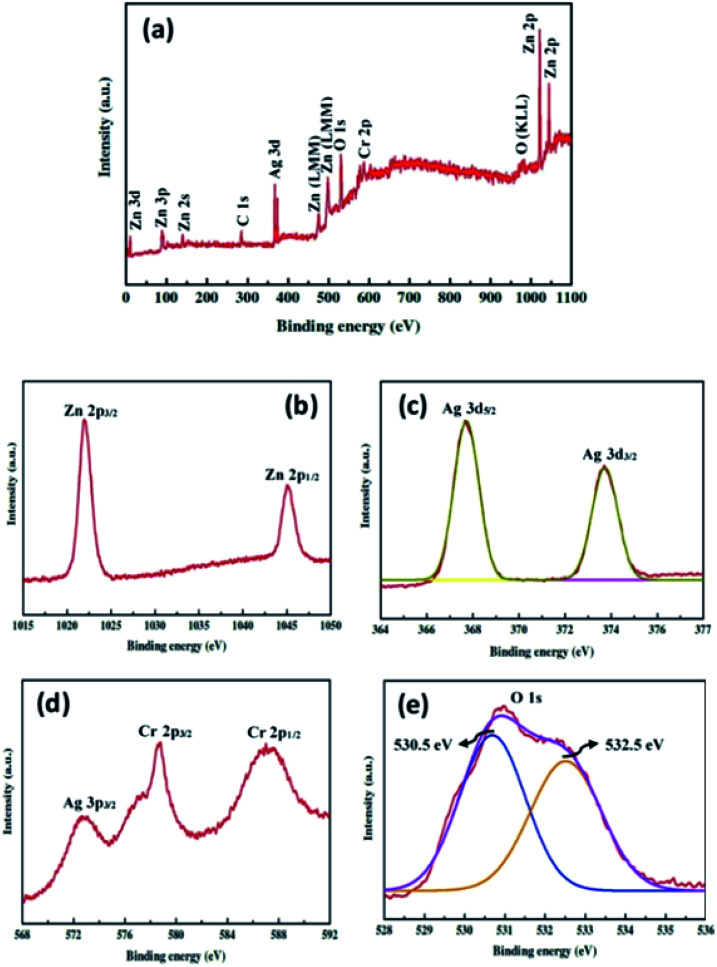
XPS spectra for the ZnO–Ag_2_CrO_4_-8 photoelectrode: (a) the survey scan and high-resolution spectra for (b) Zn 2p, (c) Ag 3d, (d) Cr 2p, and (e) O 1s.

The surface chemical composition of the ZnO–Ag_2_CrO_4_-8 photoelectrode was detected with the XPS technique. The XPS survey spectrum is seen in Fig. 4(a) and only shows Zn 2p, Ag 3d, Cr 2p and O 1s peaks without any contaminations. As shown in Fig. 4(b), the peaks centred at 1021.9 eV and 1045.1 eV correspond to the binding energies of Zn 2p_3/2_ and Zn 2p_1/2_ from ZnO.^36^ As observed in Fig. 4(c), the two major peaks at 367.6 and 373.7 eV correspond to the Ag 3d_5/2_ and Ag 3d_3/2_ orbits of Ag^+^ ions from Ag_2_CrO_4_.^37^ Meanwhile, no typical binding energies of Ag^0^ were found, which demonstrated that Ag_2_CrO_4_ is stable in the ZnO–Ag_2_CrO_4_-8 photoelectrode. As indicated in Fig. 4(d) for the chromium element, the peaks at 578.7 eV and 587.6 eV correspond to Cr 2p_3/2_ and Cr 2p_1/2_, confirming the presence of Cr^6+^.^38^ It is notable that the other peak at about 572.6 eV is assigned to the Ag 3p signal.^39^ Furthermore, Fig. 4(e) displays a slightly wide peak observed for O 1s. This peak is deconvoluted into two conspicuous peaks with binding energies of 530.5 eV and 532.5 eV, which are ascribed to the lattice oxygen and the external hydroxyl groups adsorbed on the ZnO–Ag_2_CrO_4_-8 photoelectrode surface, respectively.^40^ Therefore, it is reasonable to conclude that the XPS spectra together with XRD and EDX data strongly support that the ZnO–Ag_2_CrO_4_ heterojunctions have been successfully fabricated.

**Fig. 5:**
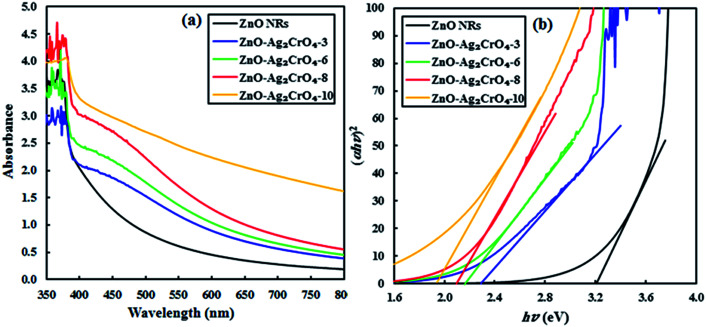
(a) UV-vis absorption spectra of the ZnO NR and ZnO–Ag_2_CrO_4_ photoelectrodes with different numbers of SILAR cycles. (b) Plots of (*αhν*)^2^*versus hν* for different samples.

UV-vis absorption spectroscopy can be performed to investigate the optical absorption capability of the fabricated photoelectrodes at different wavelengths. Fig. 5 displays the absorption spectra of pristine ZnO NR and ZnO–Ag_2_CrO_4_ heterojunctions with different SILAR cycles grown on the glass substrate. The result indicates that the spectrum of pristine ZnO NRs has a significant absorption edge at a wavelength lower than 400 nm with negligible absorption in the visible-light region, which is assigned to the intrinsic band-gap energy of pristine ZnO NRs.^12^ In addition, the spectra demonstrate that all the ZnO–Ag_2_CrO_4_ heterojunctions exhibit a broad absorption feature in the visible-light wavelengths, which is related to the visible-light absorption characteristics of Ag_2_CrO_4_ particles. Accordingly, the absorption intensity for the ZnO–Ag_2_CrO_4_ heterojunctions increases with the increase in the SILAR cycles of Ag_2_CrO_4_ on ZnO NRs. Similar phenomena were also observed by other researchers.^41–43^ In general terms, the approximate optical band-gap energies of the photoelectrodes can be obtained from the Kubelka–Munk band gap estimation theory. Based on the literature, we infer that both ZnO and Ag_2_CrO_4_ have direct transition semiconductors.^44^ Thus, as presented in Fig. 5(b), the band-gap energy (*E*_g_) has been obtained by estimating the intercept of the tangent to the Tauc's plots of (*αhν*)^2^*versus* photon energy (*hν*) to the energy axis, where *α* is the absorption coefficient, *h* is the Planck's constant, and *ν* is the light frequency. The estimated band-gap energies of all ZnO–Ag_2_CrO_4_ heterojunctions are between 1.9 and 3.2 eV. Consequently, the remarkably enhanced visible-light absorption potential of the ZnO–Ag_2_CrO_4_ photoelectrodes confirms the generation of large concentrations of e^−^/h^+^ pairs, which implies improvement in the PEC performance.

### Photoelectrochemical measurements

**Fig. 6:**
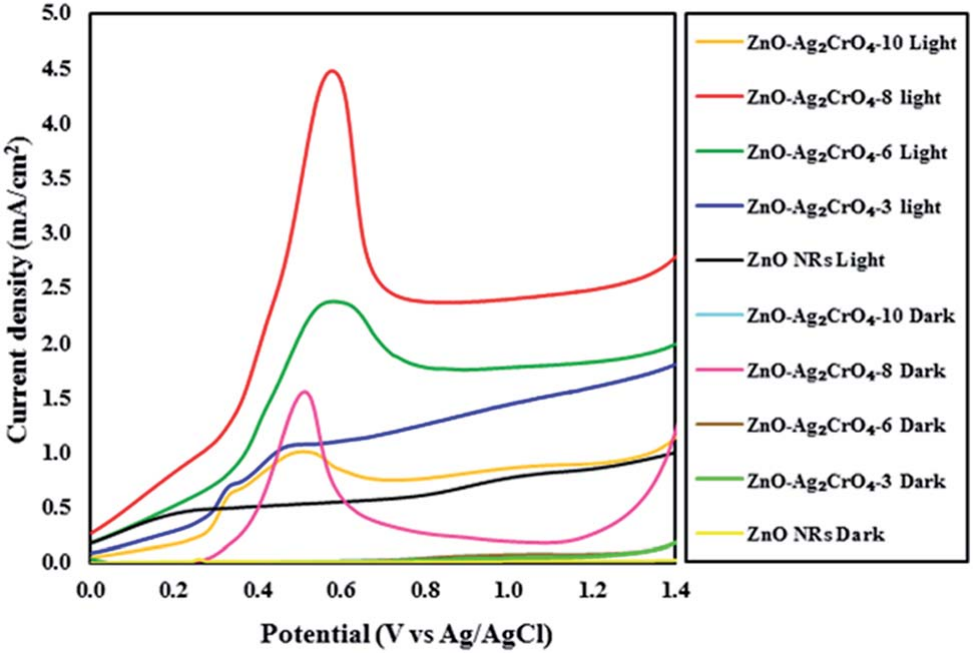
Linear sweep voltammetry curves of the ZnO NR and ZnO–Ag_2_CrO_4_ photoelectrodes with different numbers of SILAR cycles under light and dark conditions.

The activity of the photoelectrodes for the PEC cells was examined by linear-sweep voltammograms in dark and under illumination conditions from +0.0 to +1.4 V *vs.* Ag/AgCl. The characteristics of the photocurrent density *versus* the measured potential (*I*–*V* curve) for the photoelectrodes with different SILAR cycles are shown in Fig. 6. Also, the PEC performance in terms of current density is presented in Table 1. From the dark scans, we can observe that the ZnO NR photoelectrode displays a very low photocurrent density (0.02 mA cm^−2^), suggesting the good surface quality of ZnO NRs. Compared with the ZnO NR photoelectrode, the ZnO–Ag_2_CrO_4_ photoelectrodes have larger photocurrent density in the dark, indicating better electrical conductivity.

**Table 1:** Comparative study of the current density measured from the photoelectrochemical studies performed in 0.1 M Na_2_SO_4_ solution as an electrolyte, Pt wire as a counter electrode and saturated Ag/AgCl as a reference electrode at the voltage range from +0.0 to +1.4 under dark and light conditions

Photoelectrode	Dark current (*I*_dark_) mA cm^−2^	Photocurrent (*I*_light_) mA cm^−2^
ZnO NRs	0.02	0.87
ZnO–Ag_2_CrO_4_-3	0.06	1.62
ZnO–Ag_2_CrO_4_-6	0.08	1.84
ZnO–Ag_2_CrO_4_-8	0.31	2.51
ZnO–Ag_2_CrO_4_-10	0.09	0.91

A weak photocurrent was obtained for ZnO NRs upon illumination in the applied potential range, whereas a remarkably enhanced photocurrent was demonstrated for the ZnO–Ag_2_CrO_4_ heterostructures compared to that for ZnO NRs under illumination. These characteristic improvements in the photocurrent density of the photoelectrodes indicate the electrocatalytic effect of Ag_2_CrO_4_ nanoparticles on ZnO NRs at the n–n heterojunction interface, suggesting the enhancement of visible-light absorption, photogeneration and conduction of carriers in the ZnO–Ag_2_CrO_4_ heterojunction photoelectrodes. Please note that with the increase in the SILAR cycles in the ZnO–Ag_2_CrO_4_ photoelectrodes, the photocurrent increased significantly to 2.51 mA cm^−2^ for ZnO–Ag_2_CrO_4_-8. However, after further increasing the SILAR cycles up to 10, the photocurrent decreased to 0.91 mA cm^−2^ for ZnO–Ag_2_CrO_4_-10 at a bias of 1.23 V *versus* Ag/AgCl. More SILAR cycles caused the accumulation and aggregation of excess Ag_2_CrO_4_ nanoparticles on the ZnO NR surface, resulting in the destruction of the formed heterojunctions between ZnO NRs and Ag_2_CrO_4_ and finally the suppression of activity. This result implied that the ZnO–Ag_2_CrO_4_-8 heterojunction photoelectrode showed higher PEC activity and photocurrent density, which was about three times higher when compared to that of the single-component ZnO NRs. More interestingly, the photocurrent density of the ZnO–Ag_2_CrO_4_-8 photoelectrode was superior or comparable to those of some of the other reported ZnO photoelectrodes, as listed in Table 2.

**Table 2:** Characteristics of the ZnO–Ag_2_CrO_4_-8 photoelectrode along with those reported in some literatures for other heterostructure systems

Photoelectrode	Condition	Performance	Ref.
ZnO/MoS_2_	0.1 M Na_2_S buffered with H_2_SO_4_, 150 W Xe arc lamp (AM 1.5G, 100 mW cm^−2^)	930 μA cm^−2^ at 0.20 V *vs.* Hg/Hg_2_Cl_2_	31
CuFeO_2_–ZnO	0.5 M Na_2_SO_4_, pH = 6.4, visible light (*λ* > 420 nm, 10 mW cm^−2^)	58 μA cm^−2^ at 1.23 V *vs.* Ag/AgCl	45
Bi_2_S_3_/ZnO	0.1 M KOH, 250 W Xe arc lamp, (100 mW cm^−2^)	0.255 mA cm^−2^ at 0.80 V *vs.* Ag/AgCl	46
In_2_O_3_/ZnO	0.5 M Na_2_SO_4_, 300 W Xe lamp, 150 mW cm^−2^	0.4 mA cm^−2^ at 0.50 V *vs.* Ag/AgCl	47
ZnO/ZnS/Au	0.5 M Na_2_SO_4_, pH = 7.0, AM 1.5G, 50 mW cm^−2^	0.58 mA cm^−2^ at 1.00 V *vs.* Ag/AgCl	48
ZnO–Ag_2_CrO_4_-8	0.1 M Na_2_SO_4_, AM 1.5G	2.51 mA cm^−2^ at 1.23 V *vs.* Ag/AgCl	This work

**Fig. 7:**
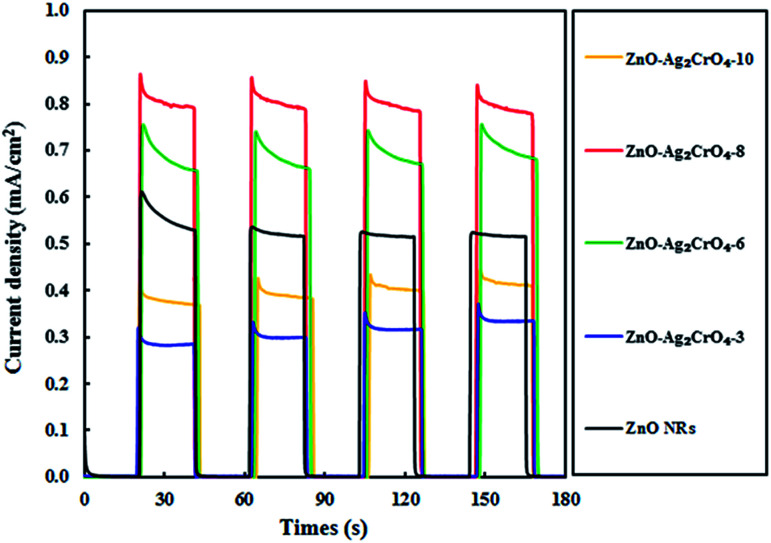
Chronoamperometry *I*–*t* curves for the ZnO NR and ZnO–Ag_2_CrO_4_ photoelectrodes with different numbers of SILAR cycles under solar-light illumination with an applied voltage of +5 V with 20 s light on/off cycles.

Undoubtedly, charge separation efficiency has a more significant role in the improvement of PEC activity. To investigate the separation abilities of the photoinduced charges, the transient photocurrent responses of the ZnO–Ag_2_CrO_4_ photoelectrodes with different numbers of SILAR cycles were measured and compared with that of the ZnO NR photoelectrode. All the tests were conducted at a certain potential of +0.5 V *versus* Ag/AgCl and the photocurrent response was recorded by switching a simulated solar light on and off with time duration of 20 s. As seen in Fig. 7, the photocurrent response of the ZnO–Ag_2_CrO_4_ photoelectrodes is higher than that of the ZnO NR photoelectrode and exhibits almost high stability after several cycles. This is in good agreement with the linear sweep voltammetry results and further demonstrates the improved performance of PEC provided by the ZnO–Ag_2_CrO_4_ heterojunctions. It is worth noting that the photocurrent enhanced with increasing SILAR cycles first, but the output decreased when the deposition cycles were increased to 10 cycles. The possible reason is that the additional Ag_2_CrO_4_ deposition led to the formation of larger aggregates around ZnO NRs, which caused the destruction of the junctions. Hence, the separation of the charge carriers in the interfaces of the heterojunction could not occur easily. Surprisingly, the saturation photoelectron current density produced in ZnO–Ag_2_CrO_4_-8 (0.80 mA cm^−2^) was about 1.54-fold higher than that in the ZnO NR photoelectrode (0.52 mA cm^−2^). These results confirmed that the n–n heterojunction formed between ZnO and Ag_2_CrO_4_ provides a wider absorption spectrum region of solar light with greater charge generation and separation, which effectively restricts the recombination of the e^−^/h^+^ pairs, leading to promoted PEC performance.

**Fig. 8:**
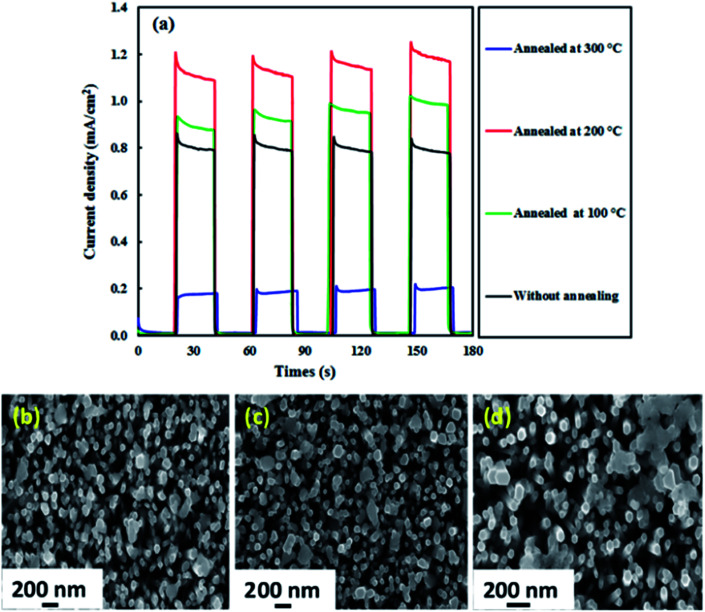
(a) Photocurrent density–time curves of the ZnO–Ag_2_CrO_4_-8 photoelectrode without annealing and annealed at 100, 200 and 300 °C at a bias potential of +0.5 V *vs.* Ag/AgCl. (b–d) FESEM images for the ZnO–Ag_2_CrO_4_-8 photoelectrode (b) without annealing, (c) annealed at 200 °C and (d) 300 °C.

The effect of annealing was also investigated because the PEC activity of photoelectrodes is usually influenced by the annealing temperature.^49,50^Fig. 8(a) displays the photocurrent results for the ZnO–Ag_2_CrO_4_-8 photoelectrode annealed at 100, 200, and 300 °C for 2 h. It is evident that the photocurrent response efficiently enhances with annealing of the photoelectrode up to 200 °C (1.20 mA cm^−2^) and then sharply decreases to 0.20 mA cm^−2^ at 300 °C. Such a ZnO–Ag_2_CrO_4_-8 photoelectrode can result from morphological changes, especially at the interface. The morphologies showing the strong effects of annealing on the photoresponse properties of the photoelectrodes annealed at 200 and 300 °C are compared in Fig. 8(c) and (d), respectively. Please note that the agglomeration and the size of the Ag_2_CrO_4_ nanostructures capped with ZnO NRs increased after increasing the annealing temperature from 200 to 300 °C. This can be clearly seen by comparing Fig. 8(b) to Fig. 8(c) and (d). It can be found that after annealing at 300 °C, the particles of Ag_2_CrO_4_ have tightly aggregated with each other, resulting in decrease in the contact surface between counterparts and destruction of the formed heterojunction at the interfaces. Hence, it was concluded that the photogenerated e^−^/h^+^ pairs could not be separated sufficiently, leading to decrease in the photocurrent in comparison with the result for the photoelectrode annealed at 200 °C (Fig. 8(c)).

**Fig. 9:**
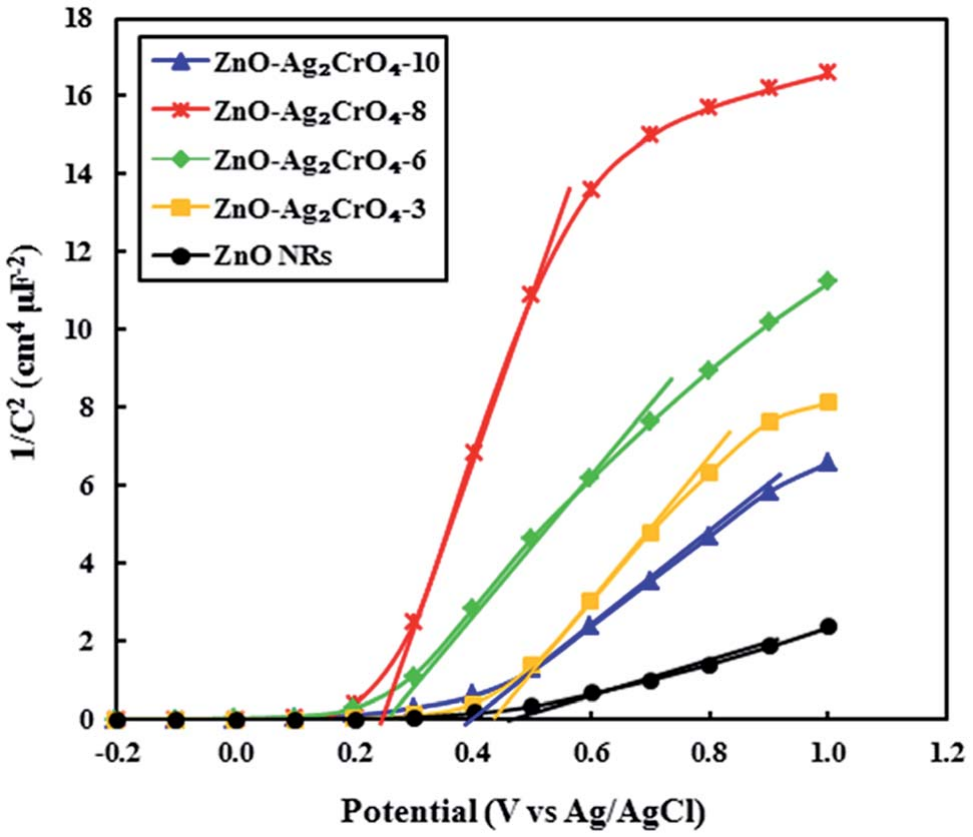
Mott–Schottky plots of 1/*C*^2^*versus* applied potential (*V*) for the ZnO NR and ZnO–Ag_2_CrO_4_ photoelectrodes with different numbers of SILAR cycles in complete darkness at a frequency of 3 kHz.

As demonstrated, the Mott–Schottky (M–S) measurement is ordinarily used in photoelectrode characterization to ascertain the carrier density and intrinsic electronic properties, which further gives quantitative information about the flat band potentials (*E*_fb_) of the as-prepared photoelectrodes.^51^ Hence, to better understand the effect of the SILAR cycles of Ag_2_CrO_4_ on the electronic proprieties of the ZnO–Ag_2_CrO_4_ heterojunctions, Mott–Schottky analysis was conducted. The M–S plots were obtained at room temperature with a frequency of 3 kHz according to the related equation. Fig. 9 shows the corresponding M–S plots for the pristine ZnO NR and ZnO–Ag_2_CrO_4_ heterojunction photoelectrodes. As seen, all of the synthesized photoelectrodes exhibit positive slopes, revealing their n-type nature as expected. Moreover, the slopes of ZnO–Ag_2_CrO_4_ heterojunctions are much larger than that of ZnO NRs, proving a major improvement in the carrier concentration after the construction of n–n heterojunctions between ZnO and Ag_2_CrO_4_ semiconductors through intimate interfacial contact. Furthermore, the extrapolation of the linear region of the slope is used to evaluate *E*_fb_ of the samples. Clearly, it is consistent with the results that *E*_fb_ of the ZnO–Ag_2_CrO_4_ photoelectrodes has nearly the same onset potential, which is smaller than that of 0.49 V *vs.* Ag/AgCl for ZnO NRs. Thus, the blue shift in *E*_fb_ is ascribed to changes in charge carrier concentration in the heterojunctions.

**Fig. 10:**
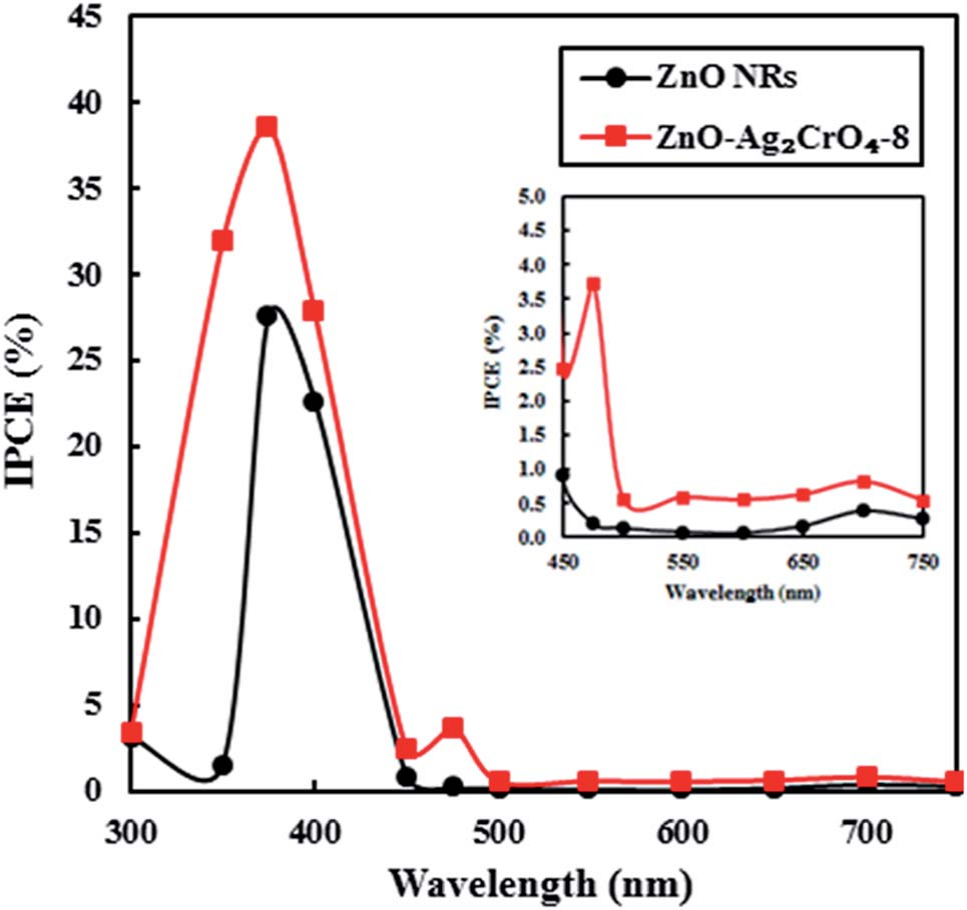
The plots of IPCE *versus* wavelength for the ZnO NR and ZnO–Ag_2_CrO_4_-8 photoelectrodes.

To examine the light-to-current conversion capacity of the ZnO NR and the ZnO–Ag_2_CrO_4_-8 photoelectrodes, the incident photon-to-current efficiency (IPCE) was studied with a monochromator light source (300–700 nm). At the same time, the generated current density was measured at each wavelength, as shown in Fig. 10. IPCE can be computed by IPCE = (1240*I*/*λJ*_light_),^2^ where *I* (mA cm^−2^), *λ* (nm) and *J*_light_ (mW cm^−2^) are the photocurrent density, wavelength, and power density of incident light, respectively. The pristine ZnO NR photoelectrode only exhibited a photoresponse at a wavelength around a maximum of 375 nm, which was comparable with its band-gap energy. Significantly, the ZnO–Ag_2_CrO_4_-8 photoelectrode showed considerable activation in the visible-light region of 450–750 nm in addition to a strong photoresponse in the UV region. Particularly, IPCE of the ZnO–Ag_2_CrO_4_-8 photoelectrode at the monochromatic wavelength of 375 nm was up to about 40%. As a consequence, the ZnO–Ag_2_CrO_4_ heterojunction can provide an effective path for photoinduced charge separation and transfer.

### Proposed mechanism

**Scheme 2:**
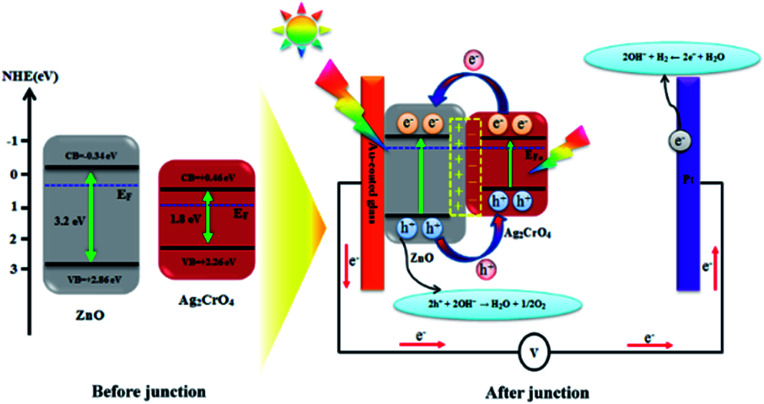
Schematic diagram showing the energy band structure and possible electron–hole separation and transportation in the ZnO–Ag_2_CrO_4_ heterojunction photoelectrodes.

Based on the above results, the improved PEC performance of the ZnO–Ag_2_CrO_4_ photoelectrode can be further explained by the construction of an n–n heterojunction between n-type ZnO and n-type Ag_2_CrO_4_ semiconductors. Hence, for a detailed understanding of the inherent mechanism of charge carrier generation, separation and transport in the ZnO–Ag_2_CrO_4_ n–n heterojunction photoelectrodes, the possible existing energy band structures are schematically exhibited in Scheme 2. The potentials of the valence band (*E*_VB_) and conduction band (*E*_CB_) for ZnO and Ag_2_CrO_4_ were calculated based on the empirical formulas^52^*E*_VB_ = *χ* − *E*^e^ + 0.5*E*_g_ and *E*_CB_ = *E*_VB_ − *E*_g_ and the results are listed in Table 3. When ZnO NRs come into contact with Ag_2_CrO_4_ nanoparticles to form an n–n heterojunction, the Fermi levels tend to align in order to attain equilibrium. Because the potential of the Fermi level in ZnO is higher than that of Ag_2_CrO_4_, the electrons in the Fermi level of ZnO migrate to that of Ag_2_CrO_4_ until the Fermi levels become coincident. Meanwhile, an inner electric field built in the interface induces the region of Ag_2_CrO_4_ to become negatively charged and the region of ZnO to become positively charged. Under simulated solar-light illumination, the band-gap excitation of the ZnO and Ag_2_CrO_4_ semiconductors occurs, creating e^−^/h^+^ pairs. Photogenerated electrons from Ag_2_CrO_4_ can move to CB of ZnO quickly with the assistance of the electric field established at the heterojunction interface. Simultaneously, holes on VB of ZnO also inject into VB of Ag_2_CrO_4_. Consequently, the inner electric field provides a spatial separation of e^−^/h^+^ pairs by accelerating the charge carrier migration across the heterojunction and restricting the e^−^/h^+^ recombination, leading to promotion in the charge carrier separation efficiency. Ultimately, the photogenerated holes can be rapidly transported to the interface between the photoelectrode and electrolyte to perform oxygen evolution, and H_2_ is produced through a reduction reaction on the Pt electrode.

**Table 3:** Calculation of the *χ*, CB, and VB potentials for ZnO and Ag_2_CrO_4_

Semiconductor	*E* ^e^ (eV) a	*χ* b	*E*_g_ (eV)	CB (eV)	VB (eV)
ZnO	4.5	5.76	3.2	−0.34	+2.86
Ag_2_CrO_4_	4.5	5.86	1.8	+0.46	+2.26

a *E* ^e^ is the energy of free electrons on the hydrogen scale.

b *χ* is the Mulliken electronegativity of the semiconductors.

## Conclusions

A facile and effective route was adopted to construct ZnO–Ag_2_CrO_4_ n–n heterojunction photoelectrodes though hydrothermal and SILAR methods for potential applications in solar-light PEC devices. The variation in SILAR cycles for Ag_2_CrO_4_ and the influence on the structural, optical, and overall PEC performances were studied. The strong absorption of the ZnO–Ag_2_CrO_4_ heterojunctions in the visible region made them promising candidates for solar-light harvesting applications. Specifically, the optimal ZnO–Ag_2_CrO_4_-8 photoelectrode presented photocurrent density of about 2.49 mA cm^−2^, which was nearly three times superior to that of the ZnO NR photoelectrode (0.85 mA cm^−2^) at 1.20 V *vs.* Ag/AgCl. In addition, the results showed that the photoelectrode annealed at 200 °C has the best activity. The photoresponse over time of the annealed photoelectrode was about 2.3-fold higher than that of the ZnO NR photoelectrode. It was concluded that the formation of the heterojunction between ZnO NRs and Ag_2_CrO_4_ particles can dramatically separate excess charge carriers and suppress the recombination of e^−^/h^+^ pairs, thereby facilitating the interparticle electron transfer at the n–n heterojunction of the ZnO–Ag_2_CrO_4_ interfaces. The photoconversion efficiency of the ZnO–Ag_2_CrO_4_-8 photoelectrode reached 40%, which was about 1.5-times that of the pristine ZnO NR photoelectrode. Based on the desirable photoelectrode structure, facile synthesis process, and promising PEC performance, this strategy might be easily extended to the fabrication of other heterojunction photoelectrode materials, which might find applications in the field of environmental and energy crises.

## Conflicts of interest

There are no conflicts of interest to declare.

## Supplementary Material
